# Internet-based self-administered intervention to reduce anxiety and depression symptomatology and improve well-being in 7 countries: protocol of a randomized control trial

**DOI:** 10.3389/fpsyg.2024.1279847

**Published:** 2024-05-07

**Authors:** Alejandro Dominguez-Rodriguez, Paulina Erika Herdoiza-Arroyo, Leivy Patricia González-Ramírez, Reyna Jazmín Martínez-Arriaga, David Villarreal-Zegarra, Antonio Carlos Santos da Silva, Joel Omar González-Cantero, Viviana Sylvia Vargas Salinas, Marinna S. Mensorio, Adrián Antonio Cisneros Hernández, Rogéria Lourenço dos Santos, Emilia Gabriela Nieto Ramos, Margarita Gabriela Albán-Terán, Joaquín Mateu-Mollá, Flor Rocío Ramírez-Martínez, Ana Marisa Colmenero Guadián, David Martínez-Rubio, Álvaro I. Langer, Claudio Araya, Rosa Olimpia Castellanos-Vargas

**Affiliations:** ^1^Department of Psychology, Health and Technology, University of Twente, Enschede, Netherlands; ^2^Faculty of Medicine, Health and Life Sciences, School of Psychology, Universidad Internacional del Ecuador, Quito, Ecuador; ^3^School of Medicine and Health Sciences, Tecnologico de Monterrey, Guadalajara, Mexico; ^4^Departamento de Clínicas de Salud Mental, Centro Universitario de Ciencias de la Salud, Universidad de Guadalajara, Guadalajara, Mexico; ^5^Instituto Peruano de Orientación Psicológica, Lima, Peru; ^6^Escuela de Psicología, Universidad Continental, Lima, Peru; ^7^Federal University of Bahia, Salvador, Brazil; ^8^Departamento de Ciencias del Comportamiento, Centro Universitario de los Valles, Universidad de Guadalajara, Ameca, Mexico; ^9^Departamento de Psicología Social, Universitat de València, Valencia, Spain; ^10^Independent researcher, São Paulo, Brazil; ^11^Health Sciences Area, Valencian International University, Valencia, Spain; ^12^Rectory, Autonomous University of Ciudad Juarez, Juarez, Mexico; ^13^Plan estratégico de Juarez, Juarez, Mexico; ^14^Department of Psychology, European University of Valencia, Valencia, Spain; ^15^Department of Nursing and Physiotherapy, University of Lleida, Lleida, Spain; ^16^Faculty of Psychology and Humanities, University San Sebastián, Valdivia, Chile; ^17^Millennium Nucleus to Improve the Mental Health of Adolescents and Youths, Imhay, Santiago, Chile; ^18^School of Psychology, University Adolfo Ibañez, Santiago, Chile; ^19^Health Sciences Department, Autonomous University of Ciudad Juarez, Juarez, Mexico

**Keywords:** randomized controlled trial, anxiety, depression, internet-based intervention, self-guided, well-being

## Abstract

**Background:**

Online psychological interventions have emerged as a treatment alternative because they are accessible, flexible, personalized, and available to large populations. The number of Internet interventions in Latin America is limited, as are Randomized Controlled Trials (RCTs) of their effectiveness and a few studies comparing their effectiveness in multiple countries at the same time. We have developed an online intervention, Well-being Online, which will be available to the public free of charge in 7 countries: Mexico, Ecuador, Peru, Chile, Brazil, Spain, and the Netherlands. We expect a reduction in depression and anxiety symptoms and an increase in well-being of the participants.

**Methods:**

A multi-country, randomized controlled trial will be conducted. The intervention is multicomponent (Cognitive Behavioral Therapy, Behavioral Activation Therapy, Mindfulness, Acceptance and Commitment Therapy, and Positive Psychology), with 10 sessions. In each country, eligible participants will be randomized to one of three groups: Enriched Intervention (interactive web design with videos, infographics, text, audio, and forum), Text Intervention (text on the website), and Wait List (control group). Repeated measures will be obtained at 5-time points. Our primary outcomes will be anxiety symptomatology, depressive symptomatology, and mental well-being. MANOVA analysis will be used for our main analysis.

**Discussion:**

This protocol describes the design of a randomized trial to evaluate the efficacy of a web-based intervention to reduce anxiety and depression symptomatology and increase subjective well-being. The intervention will be made available in four languages (Spanish, Portuguese, Dutch, and English). Its results will contribute to the evidence of effectiveness in terms of randomized trials and Internet interventions, mainly in Latin America and Europe.

## 1 Introduction

The World Health Organization (WHO) estimated that the worldwide proportion of people with depression and anxiety disorders was 4.4% and 3.6%, respectively (WHO, 2017). Anxiety and depression are as prevalent in Latin America as in other countries in the world, but access to mental health care is very limited (Pan American Health Organization, 2018). Table 1 provides an impression of how the prevalence of depression and anxiety disorders is distributed in Latin America compared to some European countries. These data suggest that depression and anxiety are as prevalent in Latin America as in European countries such as Spain and the Netherlands. In addition, due to the recent COVID-19 pandemic, these problems may have worsened (Vindegaard and Benros, 2020). Furthermore, despite previous studies reported online interventions focused mainly on developed countries, it is observed that the psychological symptoms, regardless of the country, are similar. For example, the study of Hernández-Posadas et al. (2024), which included a sample of Dutch and Mexican participants, observed similar results regarding the relationship between intolerance of uncertainty, emotional dysregulation, rumination, depression, and post-traumatic stress symptoms. However, studies like this are scarce, and more are needed.

**Table 1:** Prevalence of depression and anxiety in several Latin American and European countries (WHO, 2017).

Country	Depression (%)	Anxiety (%)
World	4.4	3.6
**Latin America**		
Mexico	4.2	3.6
Ecuador	4.6	5.6
Peru	4.8	5.7
Brazil	5.8	9.3
Chile	5.0	6.5
**Europe**		
Spain	5.2	4.1
Netherlands	4.7	6.4

By contrast, the availability of mental health care across the world shows a different pattern. The WHO Mental Health Atlas (WHO, 2021) estimated that the median number of healthcare workers per 100,000 population is 3.4, but large differences exist across the world. Europe has a median of 12.5 workers per 100,000 inhabitants, and the Americas (including the USA) of 8.6. Likewise, the number of mental hospital facilities, beds, and admissions per 100,000 population are all about 5 times as high in Europe compared to the Americas (WHO, 2021). Recent estimations suggest that the treatment gap in Latin America is big, with almost 80% of people with depressive symptoms not receiving help at all (Pan American Health Organization, 2018). In sum, it seems that the prevalence of depression and anxiety in Latin America and Europe is roughly the same, but unfortunately, this is not matched by the availability and hence accessibility to mental health care. Anxiety and depression symptomatology are often comorbid. The Netherlands Study of Depression and Anxiety showed that 67% of patients with a primary depression diagnosis had a comorbid anxiety disorder. Otherwise 63% of patients with a primary anxiety disorder also had a depressive disorder (Lamers et al., 2011). Moreover, anxiety and depression symptomatology cause a significant loss of quality of life in different areas and daily activities (Khansa et al., 2020).

Several interventions have been shown to be effective in reducing depression and anxiety symptomatology, such as Acceptance and Commitment Therapy (Bai et al., 2020; Gloster et al., 2020), Cognitive Behavioral Therapy (Butler et al., 2006), Mindfulness (Hofmann et al., 2010), Behavioral Activation for Depression (Cuijpers et al., 2007), and Positive Psychology interventions (Chakhssi et al., 2018). Such interventions are available through mental health care providers. If, as argued above, this type of care is less available or less accessible in Latin America compared to European countries, then the Latin American population is at a disadvantage. It is estimated that in Mexico, only one in 10 people who need psychological treatment have access to a mental health professional (Flores-Plata et al., 2014). This indicates the need to implement alternatives to prevent the development of these disorders and facilitate access to quality mental health services that address highly prevalent mental disorders such as depression and anxiety.

Online psychological interventions have emerged as a treatment alternative as they can be accessible, flexible, personalized, and available for large populations (Saddichha et al., 2014; Dominguez-Rodriguez and De La Rosa-Gómez, 2022). Furthermore, meta-analyses have revealed that self-applied internet interventions are effective at reducing both depression and anxiety symptomatology and increasing well-being (Pang et al., 2021; Ye et al., 2022). Specifically, the treatment components shown to be effective when applied online are Acceptance and Commitment Therapy (Thompson et al., 2021), Cognitive Behavioral Therapy (Morgan et al., 2017), Mindfulness (Spijkerman et al., 2016), and Behavioral Activation (Huguet et al., 2018), also, multicomponent proposals (Marcelle et al., 2019; Wijnen et al., 2023). Other approaches from ITLAS group have demonstrated the effectiveness of multimodal interventions in the Latino population for grief treatment (Dominguez-Rodriguez et al., 2023). However, the results of these meta-analyses are mainly based on internet interventions designed and applied in high-income countries. The number of internet interventions in Latin America is limited, as well as Randomized Controlled Trials (RCTs) about their effectiveness (Jiménez-Molina et al., 2019). The scarcity of free, scientifically based treatments in Latin America drastically impacts the quality of life of thousands of people who experience symptoms of anxiety and depression but do not yet receive treatment. Implementing a free, self-guided, online intervention would address the inequality in access to mental health support and ensure applicability in cases of a lack of trained personnel.

Adherence and dropouts are among the most discussed issues related to self-guided interventions (Cavanagh, 2010; Melville et al., 2010). It has been proposed that an enriched platform environment with more interactive elements (e.g., videos, forums, audio) will increase user engagement, adherence and reduce dropouts. Hurling et al. (2006) tested this hypothesis and found that a more interactive version of the same internet intervention to promote health behaviors (exercise) was more engaging, with fewer dropouts and better user retention indexes. A more recent study by Granet et al. (2023) obtained similar results with an interactive vs. a non-interactive group, and they also found a considerable difference in dropout rates (16% vs. 46%, respectively). Although the literature on the interactivity of internet interventions is scarce, these findings suggest that increasing the interactive elements in self-guided internet interventions may increase adherence and decrease dropouts by increasing user engagement.

The protocol proposed here has been developed by researchers from different countries on different continents for different populations, thus trying to minimize inequalities in mental health care accessibility and maximize the support and efficiency of evidence-based research. At the same time, this approach also enabled us to consider the cultural differences between the involved countries, through an adaptation of the online intervention guided by cultural sensitivity. When there is little to no therapist engagement, the cultural adaptation of mental health treatment approaches is essential (Harper Shehadeh et al., 2016). To culturally adapt our intervention, we followed the taxonomy proposed by Spanhel et al. (2021). This taxonomy includes 17 contents (e.g., characters’ appearance), methodological (e.g., design and esthetics), and procedural components (e.g., methods used to obtain information).

As we mentioned, the treatment of mental health in the world presents a disparity that needs to be solved with the application of psychological interventions in diverse cultural environments (Heim and Kohrt, 2019). For this purpose, cross-cultural studies are needed because cultural adaptation of psychological interventions is associated with greater effectiveness compared to those that are not culturally adapted (Bernal et al., 2009).

To summarize, we developed an online intervention, Well-being Online (equivalent names in different languages Bienestar Online, Bem-estar Online and Welzijn Online), which will be available to the public free of charge. It is aimed at people with mild symptoms of depression and/or anxiety and is therefore intended as a preventive tool for developing severe mental health issues. It is a co-creation of researchers from seven countries (Mexico, Ecuador, Chile, Brazil, Peru, the Netherlands, and Spain), intended to reduce the differences between those countries in the availability of interventions for depression and anxiety. The decision to focus on those seven countries is based on the previously established consortia of researchers and the scarcity of studies that compare Latin American and European samples in internet interventions. In this article, the protocol is presented. Overall, we expect (i) a decrease in depression and anxiety symptomatology and (ii) an increase in well-being. Furthermore, we will explore whether an enriched web design, with more presentation tools (videos, texts, audio) and interaction (forums), affects the intervention’s effectiveness and user engagement. We expect that an enriched web design will provoke (iii) more engagement, adherence, satisfaction, and fewer dropouts. Finally, we expect (iv) similar effectiveness between countries due to the process followed to culturally adapt the intervention.

Considering that it is a prevention program and as reported in a systematic review of programs for the prevention of depression, anxiety, and stress it was identified that the mean effect size of the programs was moderate (overall g = 0.65; Rith-Najarian et al., 2019), it is expected that the effect size of the intervention on the different study variables will be moderate.

## 2 Methods and analysis

### 2.1 Design and procedure

A multi-country (Mexico, Ecuador, Peru, Chile, Brazil, Spain, and the Netherlands), randomized controlled trial will be conducted. Our trial will be a parallel, three-arm, superiority analysis trial with two blinding levels (at the level of those delivering the intervention and those analyzing the data). In each country, eligible participants will be randomly assigned to one of three groups: (i) Enriched Intervention (EI): participants will receive the intervention with an interactive web design (videos, infographics, text, audios, forum), (ii) Text Intervention (TI): participants will receive the intervention only via text on the webpage, and (iii) Waiting List (WL): control group. Repeated measurements will be obtained for the EI and TI groups at 5-time points: M0: pre-intervention, M1: halfway intervention, M2: post-intervention, M3: 3 months follow-up, and M4: 6 months follow-up. Participants in the WL will be evaluated again once they conclude a waiting period of 30 days. If, at that assessment, the participants in the WL group still do not fulfill any exclusion criteria, then they will be assigned randomly to the EI or TI. Participants in this group will be evaluated at 6-time points: M0: pre-waiting list period, M1: After waiting list period, M2: halfway intervention, M3: post-intervention, M4: 3 months follow-up, and M5: 6 months follow-up.

### 2.2 Randomization and blinding

Participants who consent to participate, meet all the inclusion criteria, and do not fulfill any exclusion criteria will be randomly assigned to one of the three groups (Enriched Intervention, Text Intervention, and Waiting List control group) with a 1:1:1 allocation ratio stratified by country. After completing the 30 days on the waitlist, participants in the control group will be granted access to the intervention and randomly assigned to any of the two treatment arms in a 1:1 ratio. An independent researcher will perform a permuted block algorithm with random block sizes from 6, 9, or 12 allocations, using the Study Randomizer Software (2017) for all the randomization processes; with this, we ensure not to influence the order allocation and pre-randomization allocation concealment (Bogosian et al., 2015). Following these random blocks, the platform developer will give the participants access to the respective arm.

**Figure 1:**
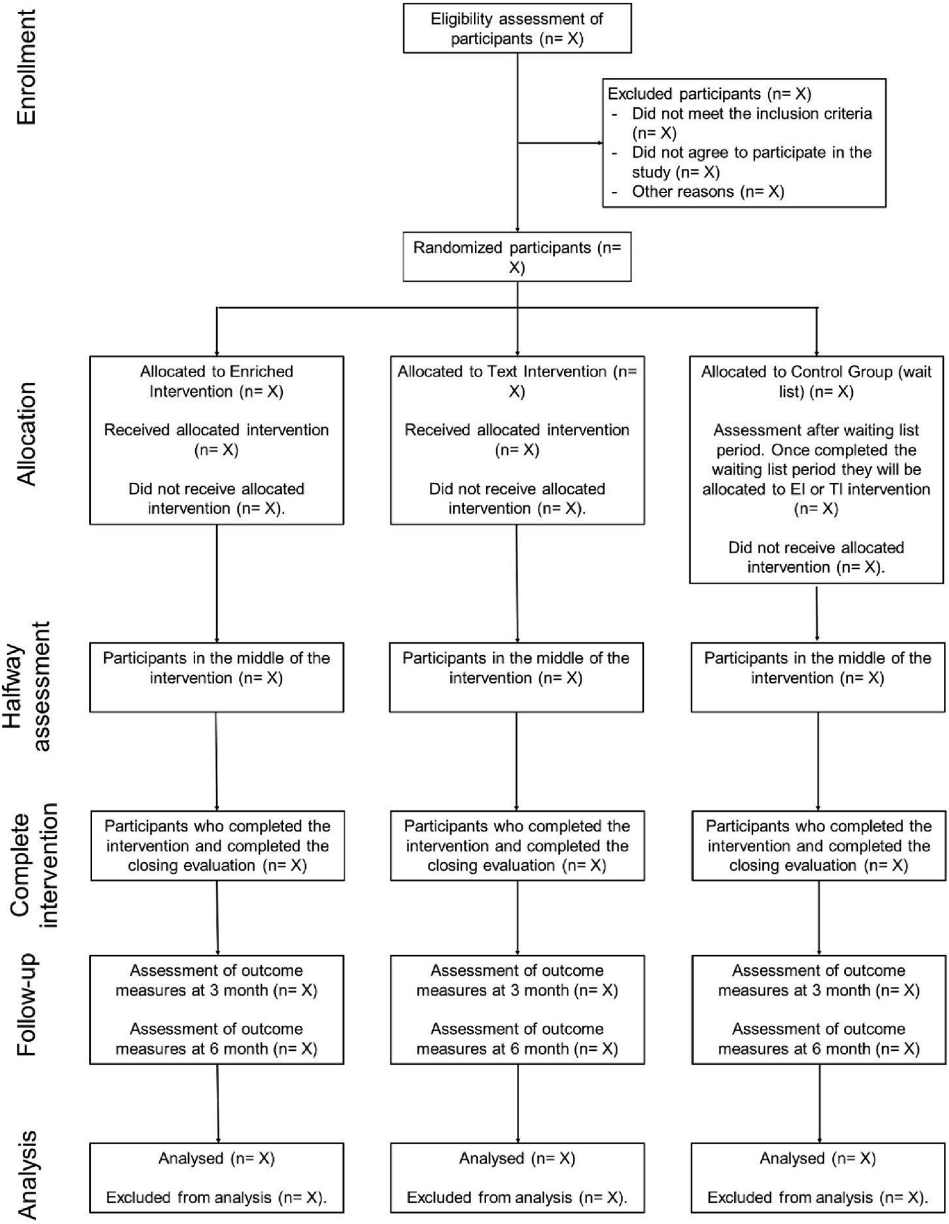
Explanatory diagram of the study of the Well-being Online Platform.

Participants will not be blinded to the intervention they receive, which is frequent among web-based interventions due to the nature of these studies (Sikorski et al., 2021; Moreno-Peral et al., 2023). Nevertheless, the researchers analyzing the data and results will remain blind to the group allocation since all analyses have been completed. The study design is shown in Figure 1.

### 2.3 Participants

#### 2.3.1 Recruitment

Digital advertising strategies will be used to invite participants. We will use digital media and social networks, particularly directed to groups or pages of health institutions on Facebook, Instagram, Twitter, and LinkedIn. In addition, the support of the traditional media will be requested to disseminate the project. Participants will be informed that the intervention is free of charge and aimed at people with mild symptoms of depression and/or anxiety.

#### 2.3.2 Selection criteria

The inclusion criteria for participation are:

- 18 years or older
- Full completion of the pre-intervention evaluation
- Residence in one of the countries participating in the study: Mexico, Peru, Chile, Brazil, Ecuador, Spain, and The Netherlands
- Fluency/proficiency in one of the intervention languages (Spanish for Mexico, Peru, Ecuador, Spain, and Chile, Brazilian-Portuguese for Brazil, English or Dutch for the Netherlands)

This intervention addresses individuals with mild depressive and/or anxiety symptomatology and includes strategies to improve resilience, adaptive coping styles and well-being. Therefore, volunteers that will be excluded are those reporting:

- Severe symptoms of depression measured by the questionnaire CES-D-R (score of 16 or higher)
- Severe symptoms of PTSD measured by the questionnaire Posttraumatic Stress Disorder Checklist for DSM-5 (score of 33 or higher)
- Severe suicidal ideation measured by the questionnaire Frequency of Suicidal Ideation Inventory (score of 11 or higher)
- To have been recently diagnosed with a depressive and/or anxiety disorder
- Currently taking medication for depressive and/or anxiety symptoms
- To have been diagnosed with another psychiatric comorbidity: personality disorder, psychotic disorder, bipolar disorder, Attention-Deficit/Hyperactivity Disorder

#### 2.3.3 Sample size

Sample size was estimated using a two-step procedure (initial estimate and simulation). We used the program Gpower 3.1 as an initial estimate for the sample size. For detecting a small effect size (0.20) with a significance level of 0.05 and a desired power of 0.80, a total number of 161 participants are required for a repeated-measures MANOVA within-between interaction for 7 countries and 5 measurements.

We then tried to confirm and extend these findings using simulations based on real data from a similar previous study conducted by Dominguez-Rodriguez et al. (2021, 2023). The data included the results of 114 participants, 45 on the treatment condition and 69 on the control condition, which after finishing a waiting period of 36 days, were assigned to the treatment condition. The participants answered the CESD-R and the GAD7 in 3 moments: (1) pre-treatment, (2) post-treatment, and (3) three-month follow-up (the 6-month follow-up was not available at the time of the calculation). The simulation was based on multilevel statistics instead of MANOVA, with two groups (fixed between-subjects factor GROUP; control vs. treatment) and three-time points (fixed within-subjects factor TIME; pre-intervention, post-intervention, and follow-up after 3 months).

We used the R statistical package v4.2.1 and the packages lme4 and simr to perform the simulations. First, the number of observations was artificially inflated from 114 to 5,000 to have enough data available for simulating a large enough range of sample sizes. Then, we performed 100 simulations each for all sample sizes between 1 and 5,000. The results confirmed the results from Gpower in that, in order to obtain a statistically significant combined GROUP and TIME effect, both on the GAD and the CESDR scores, at a significance level of 0.05 and a power of 0.80, about 160 observations would suffice. The sample sizes for the main effects of GROUP and TIME would be much lower (60 vs. 10, respectively). To be on the safe side, considering the dropout rates in previous studies of digital interventions (Moshe et al., 2022) and mental health interventions in low- and middle-income countries (Fernández et al., 2021), we estimated a 45% dropout. Thus, we aim to achieve a sample size of 232 participants for a pooled analysis of 7 countries.

### 2.4 Study groups

Participants will be randomly assigned to one of the following groups:

#### 2.4.1 Enriched intervention

The participants will have access to an enriched web platform, which will include:

- Three different information presentation types. The participants can choose to receive the information via animated videos, text, or only audio. Graphic designers created the animated videos and have an avatar—“therapist”—who explains both the intervention and exercises. We will record which medium they choose.
- A forum. The aims of the forum are: (i) increase interaction, (ii) provide peer support, and (iii) provide expert support. The participants can share their reflections, thoughts, experiences, and questions. Participants’ entries will be anonymous and filtered by a trained expert team before being made public. In the event of concerning entries (e.g., suicidal ideation-intention, adverse effects of the intervention), the participants will be contacted to provide further specialized support.

#### 2.4.2 Text intervention

The participants will have access to a web platform, including the same intervention modules as the EI group, but explained only in text form. Furthermore, they will not have access to the forum. Therefore, the EI and TI intervention groups will differ in the availability of presentation modes (EI: video, audio and/or text vs. TI: only text) and the possibility to interact (EI: forum available vs. TI: no forum available). At the end of the study, the participants in the TI group will also have access to the enriched web design.

#### 2.4.3 Waiting list control group

Participants assigned to this group will wait 30 days to be randomly assigned to either group EI or TI. During this time, they will complete the questionnaires at 2 assessment moments: M0: pre-assessment, M1: End 30 days. The 30-day period was set to consider the time that participants in the EI or TI groups would take to complete the intervention.

### 2.5 The Well-being Online intervention

Well-being Online intervention aims to reduce depressive and anxious symptoms and increase well-being. It has 10 virtual modules (or sessions) delivered online using a platform created for this purpose. This multicomponent intervention was developed based on previous studies and evidence-based psychological approaches for preventing and treating depression and anxiety disorders (Weisel et al., 2019; Coto-Lesmes et al., 2020) and for increasing well-being (Rubel et al., 2024). Psychological frameworks such as Cognitive Behavioral Therapy (CBT; Hao et al., 2023; Rubel et al., 2024), Behavioral Activation Therapy (BAT; Alirahmi et al., 2023; Hao et al., 2023), Acceptance and Commitment Therapy (ACT; Alirahmi et al., 2023; Coto-Lesmes et al., 2020), Mindfulness (Rubel et al., 2024), and Positive Psychology (Görges et al., 2018) have provided effective tools to address these therapeutic aims in previous studies and were, therefore, used in the development of the intervention contents. Table 2 presents the contents in detail.

**Table 2:** Module contents and theoretical models.

No	Modules	Theoretical model	Intervention contents
1	Understanding my feelings: anxiety and depression	CBT	Introduction to the program.
			Definitions: emotions, depressive mood, anxiety and anger.
			Functional Behavior Assessment (explained as an exercise).
			Goal setting.
2	The vicious circle of sadness	BAT	Depressive mood leads to avoidance of pleasurable activities.
			Identify activities you like (or used to like) to do.
3	Scheduling pleasant activities in your daily life	BAT	Plan pleasurable activities on your agenda.
4	What is mindfulness?	Mindfulness	Introduction to mindfulness and its beneficial effects. Exercise: conscious breathing.
5	Practicing mindfulness in my daily life	Mindfulness	Promote mindfulness practice in daily life.
			Introduction to 3 different mindfulness exercises: raisin meditation, body scanning and mountain visualization.
6	What can I do when I feel anxious?	ACT	Cognitive control is presented as a source of discomfort. Cognitive defusion and personal values exercises are performed.
7	Dealing with my emotions	CBT	Introduction to emotion regulation.
			Exercises to identify and deal with your emotions.
			Coping strategies (e.g., social support, pleasurable activities).
8	Looking for the bright side of things	Positive Psychology	Introduction to optimism and pessimism.
			Exercises to identify them both and increase optimism.
9	The strength of gratitude	Positive Psychology	Gratitude practice through three different exercises.
			Potential to increase social support.
10	How to deal with relapses	CBT and BAT	Personalized relapse prevention plan.
			End of treatment, encouragement to revisit the modules.

CBT, Cognitive behavioral therapy; ACT, Acceptance and Commitment Therapy; BAT, Behavioral activation therapy.

**Figure 2:**
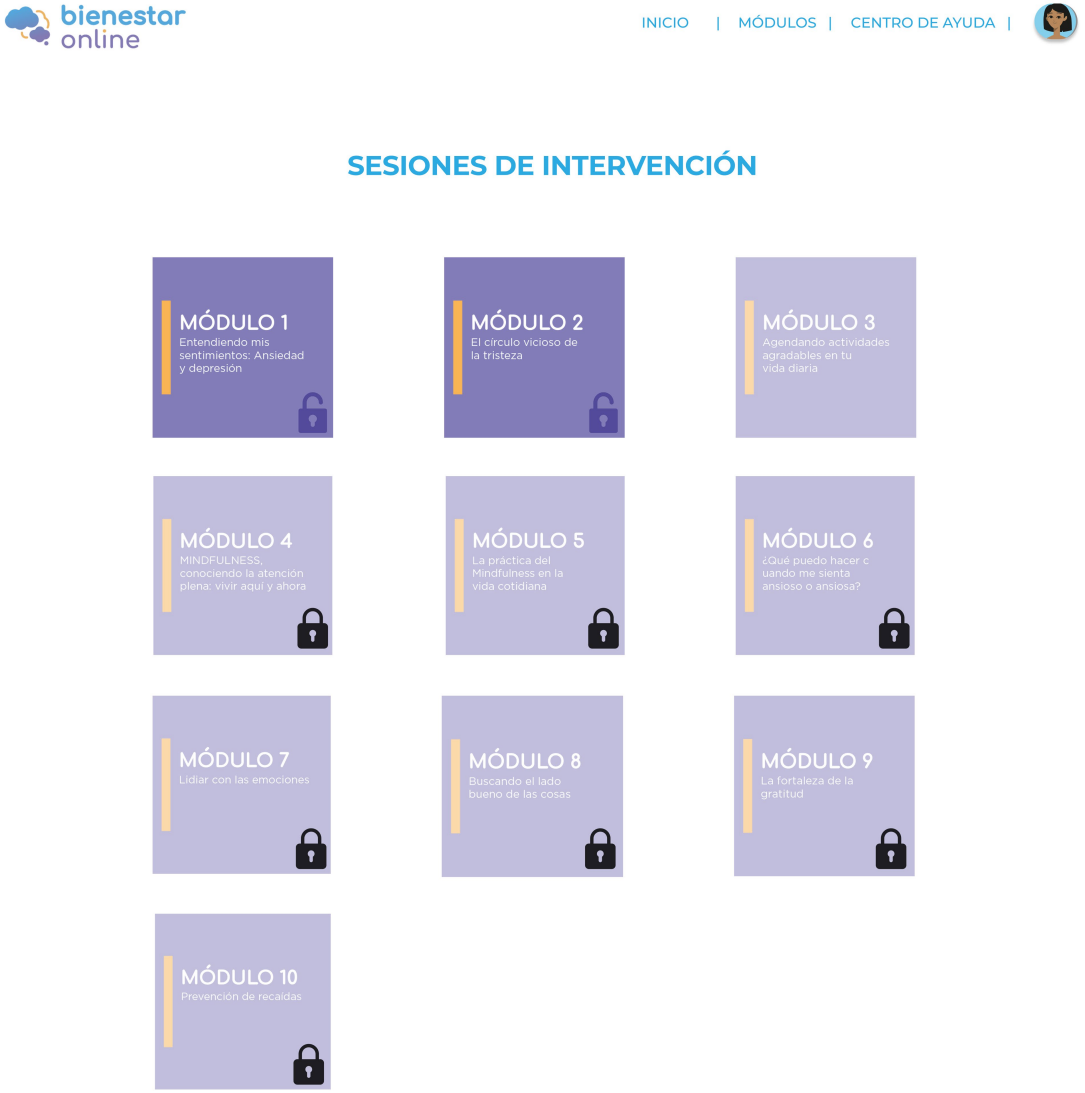
Intervention modules.

Once the participants have access to the intervention, Module 1 will be open (see Figure 2). Every time they finish a module, they must answer a quiz with 5 multiple-choice questions to evaluate their understanding of the information presented in the module. The module will be considered finished only when they correctly answer at least 3 of the 5 questions. When they finish a module, they must wait 3 days for the next module to unlock. This time between modules is intended to promote self-reflection and apply the contents of the modules to their daily life.

### 2.6 Technical guarantee of website construction

Regarding the technical solution for the Web Platform where the intervention is based, we decided to use Microsoft’s robust. NET framework, employing an object-oriented programming approach to ensure a strong and modular structure. A Structured Query Language (SQL) relational database engine was chosen to efficiently manage participants’ information, allowing accurate and secure management of internal data, statistical records and other vital information for the comprehensive operation of the platform.

Using a three-layer Model-View-Controller (MVC) structure offers several benefits in developing computational systems, especially in our platform. First, the clear separation of responsibilities between the Model (the business logic and data access), the View (the user interface) and the Controller (the management of events and requests) makes it easier to organize and maintain the code, which leads to a more orderly and scalable development. This also allows changes to be made to one layer without directly affecting the others, improving flexibility and ease of testing and debugging. Additionally, in the context of private and secure internal data contained in the platform, this architecture contributes to data security and integrity by more efficiently controlling access to and manipulation of sensitive participant information.

### 2.7 Potential adverse effects and dropout

To mitigate possible adverse effects of the intervention, participants who, at the time of the initial evaluation, will obtain high scores in clinical variables (considering the cut-off points of the instruments used) will not be able to enter the online intervention, given its preventive nature. On the contrary, those participants will be redirected through the platform to contact information for local institutions to obtain more specific and specialized help for their emotional needs.

Additionally, on the web platform on which the intervention is offered, contact information will be available to participants so they can write to team members anytime. This communication channel will be open to resolving technical issues and other concerns during the study.

Furthermore, participants will have access to the interactive forum, which content will be analyzed daily by trained personnel to detect a potential worsening of symptoms or the occurrence of adverse effects of the intervention; in this case, a member of the team will contact the person via email to do closer monitoring of the eventual deterioration of targeted symptoms and explore other potential repercussions. Monitoring adverse effects is an effective strategy for preventing them and reducing dropout rates in web-based interventions (Fenski et al., 2021). Furthermore, attrition will also be handled by notification emails sent to the participants in different intervention periods (i.e., after each completed module, when a new module is released 3 days after the completion of the previous one, and as assessment reminders).

### 2.8 Measures

Participants will be assessed through the web platform in an automated way. They will be able to answer the questionnaires at any time of the day on any day of the week. The evaluation instruments and assessment moments are presented in Table 3. We selected them using two criteria: (i) instruments most used in similar protocols and (ii) instruments validated in most countries participating in this study.

**Table 3:** Schedule of enrolment, interventions, and assessments according to the SPIRIT guidelines.

	Study period						
	Enrolment	Allocation	Post-allocation				
Timepoint	−t1	0	Pre test	Intervention	Post test	Follow-up-3	Follow-up-6
**Enrolment**							
Eligibility screen	X						
Informed consent	X						
Allocation		X					
Interventions							
Intervention group (interactive intervention)			X	X	X	X	X
Intervention group (non-interactive intervention)			X	X	X	X	X
Control group (waitlist)							
Assessments							
**Primary outcomes**							
Anxious symptoms (GAD-7)			X	X	X	X	X
Depressive symptoms (CES-D-R)			X	X	X	X	X
Mental Well-being (WEMWBS)			X	X	X	X	X
Secondary outcomes							
Stress symptoms (PSS-10)			X	X	X	X	X
Sleep quality (PSQI)			X	-	X	X	X
Experiential avoidance and psychological inflexibility (AAQ-II)			X	X	X	X	X
Life Satisfaction (SWLS)			X	X	X	X	X
Cognitive Deficits (PDQ-5)			X	-	X	X	X
Other outcomes							
Opinion treatment					X		
Client Satisfaction (CSQ-8S)					X		
System Usability Scale					X		
Telehealth Usability (TUQ)					X		
Covariates							
Sociodemographic Data			X				
Self-assessment per-module (p-m)				X			
**Exclusion variables**							
Posttraumatic Stress Disorder Checklist for DSM-5 (PCL-5)	X						
Frequency of Suicidal Ideation Inventory (FSII)	X						

#### 2.8.1 Primary outcomes

##### 2.8.1.1 Anxiety symptomatology

The General Anxiety Disorder Scale-7 (GAD-7) will be applied (Spitzer et al., 2006). This 7-item scale measures the severity of generalized anxiety disorder symptoms. Items are rated on a 4-point scale ranging from 0 (not at all) to 3 (nearly every day). The maximum total score is 21. The GAD-7 provides an assessment of anxiety for people with no symptoms (0 to 4 points), mild (5 to 9 points), moderate (10 to 14 points), and severe symptoms (15 points or more). GAD-7 has good reliability and validity in its versions in English (Spitzer et al., 2006), Spanish (Garcia-Campayo et al., 2010), Brazilian-Portuguese (Moreno et al., 2016), and Dutch (Donker et al., 2011).

##### 2.8.1.2 Depressive symptomatology

The Center for Epidemiologic Studies Depression Scale will be applied in its revised version (CESD-R; Eaton et al., 2004). This 20-item scale measures symptoms of depression, covering the criteria of temporality established in the DSM-IV. Items are rated on a 5-point scale ranging from 0 (not at all or less than 1 day) to 4 (nearly every day for 2 weeks). The total score is calculated as a sum of the 20 questions, transforming the values for the top two response options (i.e., responses rated as 3 and 4) each with a score of 3. Total scores range from 0 to 60. A cutoff score of 16 points or more suggests symptoms of depression. CESD-R has good reliability and validity in its versions in English (Eaton et al., 2004), Spanish (González-Forteza et al., 2011), Brazilian-Portuguese (Faro et al., 2020), and Dutch (Beekman et al., 1997).

##### 2.8.1.3 Mental well-being

The Warwick-Edinburgh Mental Well-Being Scale (WEMWBS) will be applied (Tennant et al., 2007). This 14-item scale measures mental well-being and includes hedonic (i.e., affects, life satisfaction) and eudemonic (i.e., positive relationships, psychological functioning) items. Items are rated on a 5-point scale ranging from 1 (not at all) to 5 (all the time). The total score ranges from 14 to 70. The higher the score, the higher the mental well-being (Tennant et al., 2007). WEMWBS has good reliability and validity in its versions in English (Tennant et al., 2007), Spanish (Carvajal et al., 2015), Brazilian Portuguese (Santos et al., 2015), and Dutch (Ikink et al., 2012).

#### 2.8.2 Secondary outcomes

##### 2.8.2.1 Perceived stress

The Perceived Stress Scale (PSS-10) will be applied (Cohen et al., 1983). This 10-item scale measures the level of stress perceived in the last month. Items are rated on a 5-point scale ranging from 0 (never) to 4 (very often). The higher the score, the higher the stress level. PSS-10 has good reliability and validity in its versions in English (Denovan et al., 2019), Spanish (González-Ramírez et al., 2013), Brazilian Portuguese (Siqueira Reis et al., 2010), and Dutch (van Eck et al., 1996).

##### 2.8.2.2 Sleep quality

The Pittsburgh Sleep Quality Index (PSQI) will be applied (Buysse et al., 1991). This 19-item scale measures a wide variety of factors relating to sleep quality. Items combine to form 7 components: sleep duration, sleep disturbance, sleep latency, daytime dysfunction, sleep efficiency, overall quality of sleep, and use of sleep medication (Buysse et al., 1989), which are rated considering the frequency or severity of sleep disturbance using scores ranging from 0 (not during the past month) to 3 (three or more times a week). The total score of the instrument is obtained by the sum of the component scores ranging from 0 to 21, with higher scores representing lower sleep quality PSQI has good reliability and validity in its versions in English (Buysse et al., 1991), Spanish (Jiménez-Genchi et al., 2008), Brazilian Portuguese (Bertolazi et al., 2011) and following Zwart et al. (2018), the Dutch version provided by eProvide® will be used.

##### 2.8.2.3 Avoidance and inflexibility

The Action Acceptance Questionnaire II (AAQ-II) will be applied (Bond et al., 2011). This 7-item scale measures experiential avoidance and psychological inflexibility. Items are rated on a 7-point scale ranging from 1 (never true) to 7 (always true). It is one of the most widely used scales in evaluating acceptance and commitment-based web therapies (Thompson et al., 2021). AAQ-II has good reliability and validity in its versions in English (Bond et al., 2011), Spanish (Ruiz et al., 2013), Brazilian Portuguese (Barbosa and Murta, 2015), and Dutch (Jacobs et al., 2008).

##### 2.8.2.4 Life satisfaction

The Satisfaction with Life Scale (SWLS) will be applied (Diener et al., 1985). This is a short scale of 5 items answered with a Likert format (1: strongly disagree to 7: strongly agree). The scores range from 5 to 35, with higher scores indicating greater life satisfaction. SWLS has good reliability and validity in its versions in English (Diener et al., 1985), Spanish (López-Ortega et al., 2016), Brazilian Portuguese (Zanon et al., 2014), and Dutch (Arrindell et al., 1999).

##### 2.8.2.5 Affect

The Positive and Negative Affect Schedule (PANAS) will be applied (Watson et al., 1988). This 20-item scale measures emotional states with two outcomes: strength of negative affect and strength of positive affect. Items are rated on a 5-point scale ranging from 0 (not at all) to 4 (extremely). PANAS has good reliability and validity in its versions in English (Watson et al., 1988), Spanish (Ortuño-Sierra et al., 2015), Brazilian Portuguese (De Carvalho et al., 2013), and Dutch (De Engelen et al., 2006).

##### 2.8.2.6 Perceived cognitive deficits

The Perceived Deficits Questionnaire (PDQ-5) will be applied (Sullivan et al., 1990). This 5-item scale measures perceived cognitive deficits associated with depressive-type disorders. Items are rated on a 5-point scale ranging from 1 (rarely) to 5 (always). PDQ-5 has good reliability and validity in its versions in English (Sullivan et al., 1990). Unfortunately, the instrument is not available in Spanish, Brazilian Portuguese, nor Dutch. Therefore, the researchers will translate it, and the instruments will be validated in the remaining countries.

#### 2.8.3 Other outcomes

##### 2.8.3.1 Satisfaction with the intervention

Two instruments will be used to assess satisfaction with the intervention. The Opinion Treatment is a 6-item scale measuring (i) satisfaction with the treatment, (ii) whether they would recommend the treatment to a friend or family member, (iii) whether the user considers the treatment useful, and (iv) whether they believe the treatment was difficult to manage or aversive. This scale is explained in more detail in Botella et al. (2009). The second instrument is the Client Satisfaction Questionnaire (CSQ-8; Larsen et al., 1979). This 8-item scale measures the level of satisfaction with the treatment and has been used to evaluate self-administered treatments before and showed a high degree of internal consistency (De Brey, 1983; Roberts and Attkisson, 1983).

##### 2.8.3.2 System usability

Two instruments will be used to assess system usability, and the System Usability Scale will be applied (see Brooke, 1996). It is composed of 10 items, rated on a 5-point scale ranging from 0 (disagree) to 4 (completely agree) and is an instrument designed to validate the usability of a system. The Telehealth Usability Questionnaire will be used to assess the usability of telehealth systems through two dimensions: 1) effectiveness and 2) ease of use. TUQ has 12 items rated on a 7-point scale ranging from 1 (disagree) to 7 (agree) (TUQ, see Parmanto et al., 2016).

#### 2.8.4 Covariates

##### 2.8.4.1 Sociodemographic data

Patient confidentiality will be the priority, and data such as name, address, and telephone number will not be requested at any time. However, other relevant data will be collected (such as education, employment status, marital status, age, gender, city of residence, substance use, presence of mental disorders or being in psychological treatment), to comply with the points stipulated by the inclusion and exclusion criteria. Other data will be asked, such as whether the person lives alone or with someone.

##### 2.8.4.2 Self-assessment per module

At the end of each module, a self-assessment composed of 5 items with multiple options formats will be conducted, in which self-exploration questions related to the information reviewed throughout the module sessions will be asked. These questions will serve as a self-assessment of the knowledge acquired, introducing the possibility of reviewing the module (videos, infographics) in case the number of correct answers is insufficient. The participants must answer at least 3 of 5 questions correctly to proceed to the further module.

### 2.9 Exclusion outcomes

Two instruments will be used to assess two exclusion criteria in this study. Severe PTSD symptomatology will be assessed with the Posttraumatic Stress Disorder Checklist for DSM-5 (PCL-5, Weathers et al., 2013), and severe suicidal ideation will be assessed with the Frequency of Suicidal Ideation Inventory (FSII, Chang and Chang, 2016). Both instruments are described elsewhere (see Weathers et al., 2013 and Chang and Chang, 2016) and show adequate reliability and validity indicators in the countries involved in this study.

### 2.10 Data analysis

#### 2.10.1 Description of participants

We will perform a descriptive analysis of proportions for the categorical variables and an analysis of central tendency and dispersion for the continuous variables and outcomes. Also, we will perform the analysis for each measurement (pre-test, intervention, post-test, follow-up-3, follow-up-6) and each of the three groups. Following a conservative approach, we will use only valid data from participants who completed the intervention or at least arrived at half of the intervention since an assessment was also implemented there. However, only participants that completed the 10 sessions will be considered as completers.

#### 2.10.2 Assessment of immediate treatment effects (main analysis)

The investigators will conduct a -blind data analysis. All analyses will use two-tailed hypothesis testing. A between-subjects MANOVA will be used to test for between-group differences in the degree of change between outcomes. Unlike some ANOVAs, MANCOVA takes into account multivariate relationships between anxiety symptoms, depressive symptoms, and other outcomes. We will use Levine’s test of equal error variance to test the assumption of homogeneity of variance, which indicates the robustness of the multivariate analysis. MANOVA analysis will be used, considering the seven country groups and using three measures (pre-test, middle of the intervention, and post-test) for the participants initially assigned to one of the two intervention groups. For the participants in the control group, the same analysis will be conducted, however, with four measures (pre-test, post-waiting list period, middle of the intervention, and post-test). The analysis is conducted on an intention-to-treat basis to avoid overestimation of clinical effectiveness and to control for dropout bias. Missing responses will be imputed using multiple imputations (Sterne et al., 2009). The models will be adjusted based on the three measures and the variables gender and age. The adjusted partial eta squared (adj η2) will be used to evaluate the effect size, knowing that the values 0.02 indicate a small effect size, 0.15 medium, and 0.30 large (Mordkoff, 2019). Data from this study will be analyzed using SPSS v.26 and STATA 17.

#### 2.10.3 Assessment of treatment sustainability

A Mixed Model ANOVA will be conducted by the seven country groups and by time (pre-test, intervention, post-test, follow-up-3, and follow-up-6) to assess the effect of the three intervention groups. In addition, the adjusted partial eta squared (adj η2) will be used as the effect size, and an intention-to-treat analysis will be used.

## 3 Discussion

The present research protocol has three main goals. First, to assess the effectiveness of a free self-guided internet intervention (Well-being Online) designed to reduce depression and anxiety symptomatology and increase well-being. Second, considering that the intervention has been culturally adapted for its application in different countries in Latin America and Europe, this study will compare access, adherence, user experience, and effectiveness between these countries. And third, the study aims to examine the effect of interactive elements, such as forums, presentation forms, videos, audios, and text, on the intervention’s adherence, dropout, and effectiveness. It is expected that Well-being Online will significantly reduce levels of depression and anxiety and that users will report high levels of usability and satisfaction with the intervention.

For ethical considerations, the project ensured the participation of all subjects in any intervention modalities. Within the comparison groups, the presentation modality, interactivity levels, and waiting time were systematically manipulated. This design enabled the observation of the anxiety and depression symptoms among participants devoid of any intervention. The findings of the study could demonstrate whether a more interactive version of online intervention contributes to enhanced efficacy.

Well-being Online, is a multi-component intervention, with strategies based on five therapy components: cognitive behavioral therapy, behavioral activation therapy, mindfulness, acceptance and commitment therapy, and positive psychology. Therefore, it is an evidence-based intervention, as all of these components have shown high effectiveness in treating anxiety and depression previously (Carr and Finnegan, 2015; Bai et al., 2020).

Similar Internet-based self-guided interventions to reduce depressive and anxious symptomatology have shown promising results (Taylor et al., 2021; Kurniawan et al., 2022). However, Well-being Online will be provided at no cost across seven distinct countries to improve the availability and accessibility of mental health therapies in regions with limited access to mental health care and where the cost of services is prohibitively high, so it can be beneficial for people who cannot afford traditional psychological treatment (Bretón-López et al., 2017). This is especially pertinent in Latin American countries, where mental health care is often scarce and difficult to afford (Jiménez-Molina et al., 2019). Therefore, the design of preventive interventions is also useful in reducing psychological and psychiatric disorders.

One of the primary benefits of a self-guided intervention is the convenience it offers. Individuals can access the intervention from the comfort of their homes, at a time that works best for them, without needing to schedule and attend appointments with a mental health professional (Andersson and Titov, 2014).

By integrating mindfulness and positive psychology, the intervention addresses negative symptoms and promotes positive emotions and behaviors, so it takes a holistic approach to mental health (Shafran and Bennett, 2017). For example, a previous study showed a negative association between one’s difficulty in identifying feelings and his experience of feelings of compassion and concern toward others (Diotaiuti et al., 2021). Another strength of Well-being Online is its self-guided nature because there is evidence of the efficacy of this type of interventions, which aim to empower individuals in their recovery by providing them with the skills and confidence they need to recognize and manage their health problems (Lean et al., 2019).

The participation of the seven countries has led to cultural adaptations in language and materials, following the recommendations of the literature on this topic (Spanhel et al., 2021). Well-being Online will be available in four languages (Spanish, Portuguese, Dutch, and English) and seven countries (Mexico, Ecuador, Peru, Chile, Brazil, Spain, and the Netherlands), so a relatively unlimited number of people can access it. The decision to make the contents available in English for the population in the Netherlands is because many people live in that country for studies or work in Dutch but speak fluent English. Some studies indicate that 90% of the population claim to converse in English (Eurobarometer, 2006; Yao and van Ours, 2015). Also, according to the organization Education First English Proficiency Index, for the fifth year in a row, the population in the Netherlands has been recognized as the best country with a proficiency of very in the ranking of 2.2 million of adults in 113 countries and regions (Education First English Proficiency Index, 2023). On the other hand, although Spain and Netherlands are classified as developed countries, free accessible online interventions are still not widespread, and many people cannot access them.

Additionally, although technology is successfully used in psychology research (Mancone et al., 2023), few studies evaluate the acceptability and feasibility of the online treatment (Tur et al., 2021), which would allow making the necessary adjustments to ensure the intervention’s effectiveness. These two elements represent strengths in the design of Well-being Online. Furthermore, if the study results are positive, this intervention could be extended to other Latin American and European countries.

Well-being Online will compare the EI vs. TI, and WL. In addition to this, animated videos, an online forum, and exercises embedded on the web page will offer users an interactive and enjoyable platform that not only allows them to obtain information but also to put intervention techniques into practice and interact with peers and professionals to receive feedback and promote treatment adherence. Beyond, adherence monitoring will be done through the web page, identifying through usage statistics, the number of times the participants access to the platform, the days they log in, and whether they access the intervention sessions; this will allow sending reminders via email to those users in which there is a delay with the intervention, to invite them to resume the sessions.

The multiple evaluations carried out during the intervention will also provide estimated data on anxiety, depression, stress, sleep quality, experiential avoidance and psychological inflexibility, satisfaction with life, mental psychological well-being, perceived deficits, post-traumatic stress and suicidal ideation, on users in different countries, which can also be compared by the sociodemographic variables collected. Furthermore, although the number of questionnaires implemented in different parts of the study could be perceived as a high number, we ensured to select questionnaires that are short and widely validated, furthermore previous studies conducted by the team members (Dominguez-Rodriguez et al., 2023; Martínez-Arriaga et al., 2023) have found that participants do answer all the assessments, and this helps to analyze the impact of the intervention.

This protocol should be considered under the following limitations. First, the intervention relies on individuals self-selecting to participate; this means that those who are more motivated to seek help may be more likely to participate, potentially skewing the results toward more positive outcomes. Higher motivation and different characteristics of voluntary participants have already been discussed in other studies (Amarnath et al., 2023). Second, it will have limited generalizability, due to the study may not be representative of the wider population. It may only include individuals who have access to and are comfortable using internet-based interventions. According to data from the World Bank, in Latin America, approximately 74% of the population has Internet access (The World Bank, 2023). However, this does not reflect people with limitations in technological skills that could restrict online intervention use. Third, the assessment tools used may not capture the full range of mental health outcomes or may not be sensitive enough to detect changes in symptom severity. However, the use of standardized tests has been prioritized as the main variables of this study. Fourth, there exists a lack of personalization, meaning that the intervention may not be tailored to individual needs and preferences, which could limit its effectiveness for certain individuals (McAllister-Williams et al., 2020). Fifth, the effectiveness of the intervention may depend on the individual’s ability to adhere to the protocol, and it is unclear how many participants will complete the program as designed. It has been noted in other studies that there are often difficulties in engaging and retaining participants long enough or in adhering to established protocols (Jiménez-Molina et al., 2019). Non-adherence may reduce the overall effectiveness of the intervention. Sixth, technical issues, such as internet connectivity problems or issues with the platform, may affect the ability of participants to access and use the intervention. Seventh, the intervention does not involve direct interaction with a therapist, which may limit its effectiveness compared to interventions that include therapist support (Jiménez-Molina et al., 2019). Finally, one additional limitation of this study is that the intervention platform does not offer an option to identify users who already entered the intervention but were dissatisfied with the treatment modality randomly assigned and, after abandoning it, reenter the intervention. Future studies should address this issue, for example, by incorporating an IP identifier for participants who sign in multiple times with different email accounts to automatically assign them every time to the same study arm (Sikorski et al., 2021).

## 4 Conclusion

Well-being Online pretends to decrease anxiety and depression symptomatology and increase subjective well-being. It will be available in four languages (Spanish, Portuguese, Dutch, and English) and seven countries (Mexico, Ecuador, Peru, Chile, Brazil, Spain, and the Netherlands). By conducting a multi-country, randomized controlled trial, this protocol aims to contribute valuable insights into the effectiveness of such interventions across diverse cultural contexts. The inclusion of seven countries in this study, in addition to the potential to replicate this study in other countries, highlight the commitment to making mental health resources accessible on a global scale. Besides, the development and implementation of the Well-being Online intervention represent a significant step forward in addressing the limited availability of online psychological interventions, mainly in Latin America and Europe.

Its methodological approach and rigorous measures through repeated assessments at multiple time points, enhances the robustness of its potential findings. Anticipating positive outcomes, including a reduction in depression and anxiety symptoms and an improvement in overall well-being among participants, we envision that the Well-being Online intervention could serve as a model for future online mental health initiatives. The results of this study hold the potential to inform and shape the landscape of accessible and effective mental health interventions, not only in the participating countries but also beyond. The commitment to providing free access to the intervention would help to democratizing mental health support and fostering well-being across diverse populations.

## Ethics statement

The study counts currently with the approval of the Ethics Committee of the Universidad Autónoma de Ciudad Juárez, Mexico (CEI-2022-2-761), the Universidad Europea de Valencia, Spain (CIPI/23.084), the Fundação de Ensino e Pesquisa em Ciências da Saúde, Brazil (CAAE: 65631922.3.0000.5553) and the Universidad César Vallejo, Peru (001-CEI-EPM-UCV-2024). At the moment of this publication, the rest of the countries are pending to receive the ethics committee approval. The trial was registered with the ClinicalTrials.gov (NCT05443139).

## Author contributions

AD-R: Writing – review & editing, Writing – original draft, Validation, Supervision, Resources, Project administration, Methodology, Investigation, Funding acquisition, Conceptualization. PH-A: Writing – review & editing, Writing – original draft, Validation, Supervision, Methodology, Investigation, Funding acquisition, Data curation, Conceptualization. LG-R: Writing – review & editing, Writing – original draft, Supervision, Resources, Project administration, Investigation, Funding acquisition, Conceptualization. RM-A: Writing – review & editing, Writing – original draft, Supervision, Project administration, Investigation, Funding acquisition, Conceptualization. DV-Z: Writing – review & editing, Writing – original draft, Supervision, Resources, Methodology, Investigation, Funding acquisition, Formal Analysis, Data curation. AS: Writing – review & editing, Supervision, Resources, Project administration, Methodology, Investigation, Funding acquisition. JG-C: Writing – review & editing, Writing – original draft, Supervision, Resources, Project administration, Methodology, Investigation, Funding acquisition, Conceptualization. VV: Writing – review & editing, Resources, Project administration, Investigation, Funding acquisition, Conceptualization. MM: Writing – original draft, Supervision, Resources, Project administration, Investigation, Funding acquisition, Conceptualization. ACH: Writing – original draft, Supervision, Resources, Project administration, Methodology, Investigation, Funding acquisition, Conceptualization. RL: Writing – review & editing, Supervision, Resources, Project administration, Funding acquisition, Conceptualization. EN: Writing – review & editing, Writing – original draft, Resources, Project administration, Investigation. MA-T: Writing – review & editing, Writing – original draft, Resources, Project administration, Investigation. JM-M: Resources, Methodology, Investigation, Formal Analysis, Conceptualization, Writing – original draft. FR-M: Writing – review & editing, Supervision, Resources, Project administration, Investigation, Funding acquisition. ACG: Conceptualization, Writing – review & editing, Supervision, Resources, Project administration, Investigation, Funding acquisition. DM-R: Writing – review & editing, Supervision, Resources, Project administration, Investigation, Funding acquisition. ÁL: Writing – review & editing, Supervision, Resources, Project administration, Investigation, Funding acquisition. CA: Writing – review & editing, Supervision, Resources, Project administration, Investigation, Funding acquisition. RC-V: Writing – original draft, Methodology, Conceptualization, Project administration, Investigation, Funding acquisition.
